# Transient Suppression of Dbx1 PreBötzinger Interneurons Disrupts Breathing in Adult Mice

**DOI:** 10.1371/journal.pone.0162418

**Published:** 2016-09-09

**Authors:** Nikolas C. Vann, Francis D. Pham, John A. Hayes, Andrew Kottick, Christopher A. Del Negro

**Affiliations:** Department of Applied Science, The College of William and Mary, Williamsburg, Virginia, United States of America; Baylor College of Medicine, UNITED STATES

## Abstract

Interneurons derived from Dbx1-expressing precursors located in the brainstem preBötzinger complex (preBötC) putatively form the core oscillator for inspiratory breathing movements. We tested this Dbx1 core hypothesis by expressing archaerhodopsin in Dbx1-derived interneurons and then transiently hyperpolarizing these neurons while measuring respiratory rhythm in vitro or breathing in vagus-intact adult mice. Transient illumination of the preBötC interrupted inspiratory rhythm in both slice preparations and sedated mice. In awake mice, light application reduced breathing frequency and prolonged the inspiratory duration. Support for the Dbx1 core hypothesis previously came from embryonic and perinatal mouse experiments, but these data suggest that Dbx1-derived preBötC interneurons are rhythmogenic in adult mice too. The neural origins of breathing behavior can be attributed to a localized and genetically well-defined interneuron population.

## Introduction

Central pattern generator (CPG) networks produce neural activity that underlies rhythmic motor behaviors such as walking, swimming, chewing, and breathing. The CPG for inspiratory breathing movements resides in the preBötzinger Complex (preBötC) of the ventral medulla [1,2], but its cellular composition in adult mammals remains incompletely understood.

Efforts to classify the cellular core of the preBötC have focused on peptide and peptide receptor-expressing, as well as glutamatergic, brainstem interneurons [3–9]. Silencing or killing peptide and peptide receptor-expressing neurons causes severe respiratory pathology as well as long-lasting apnea in adult rats [4,9,10]. In addition, excitatory synaptic communication mediated by AMPA receptors is essential for rhythmogenesis and respiratory motor output in in vitro breathing models [11,12]. Mice lacking the vesicular glutamate transporter VGLUT2 fail to breathe, even though the preBötC forms, because its constituent rhythmogenic neurons do not activate and synchronize [8].

These competing classification schemes may converge in one genetic class of brainstem interneurons whose precursors express the homeodomain transcription factor Dbx1 (hereafter referred to as Dbx1 neurons). When studied at perinatal stages of development, Dbx1 preBötC neurons express the same peptides and peptide receptors described above and are overwhelmingly glutamatergic. The commissural axons of Dbx1 preBötC neurons synchronize embryonic respiratory rhythms, and Dbx1 knock-out mice die at birth of asphyxia [13–15]. Moreover, the selective laser ablation of Dbx1 preBötC neurons in a neonatal slice model of breathing degrades and decelerates inspiratory-related motor output until irreversible rhythm cessation [16]. Therefore, we, and others, proposed the Dbx1 core hypothesis [14,15,17], which posits that Dbx1 neurons comprise the core CPG for inspiratory breathing movements. As recounted above, accumulating evidence suggests that Dbx1 preBötC neurons are rhythmogenic at perinatal stages of development. Regarding their role in adults, Koizumi *et al*. [18] transiently inhibited Dbx1 preBötC neurons in rhythmically active in situ preparations from adult mice and reported changes in the frequency of respiratory motor output corresponding to the strength of optogenetic inhibition. Further, using vagus-intact adult mice in vivo, Cui *et al*. found that stimulating Dbx1 preBötC neurons via channelrhodopsin could evoke inspiratory motor bursts during the expiratory cycle [19]. These in situ cell-silencing experiments [18] coupled with the in vivo stimulation experiments [19] further support the Dbx1 core hypothesis. Nevertheless, an important test yet to be performed is to silence or diminish the function of Dbx1 preBötC neurons in intact adult mice.

Here we test the Dbx1 core hypothesis by activating the proton pump archaeorhodopsin-3 (Arch) in Dbx1 interneurons while observing breathing behavior in vagus-intact adult mice as well as in vitro models of the behavior. Photoinhibition impedes fictive breathing, and breathing movements, up to and including complete cessation of the (fictive) behavior. Whereas Dbx1-derived interneurons were previously studied in the context of embryonic and early neonatal development, these results provide additional evidence that Dbx1 preBötC neurons are rhythmogenic in adult mice as well. Therefore, we now understand both the site (preBötC) for inspiratory rhythm generation and have further confidence regarding the neuron class (Dbx1-derived) responsible for rhythmogenesis in adult as well as perinatal rodents.

## Materials and Methods

### Mice

The Institutional Animal Care and Use Committee at the College of William and Mary approved these protocols. We used female mice that express Cre recombinase fused to a tamoxifen-sensitive mutant form of the human estrogen receptor (CreER^T2^) in cells that express *Dbx1*, i.e., *Dbx1*^*CreERT2*^ [20]. These mice were mated with male Ai35D reporter mice whose *Rosa26* locus was modified by targeted insertion of a *LoxP*-flanked STOP cassette followed by a fusion gene coding for Arch and enhanced green fluorescent protein (EGFP) [21]. Tamoxifen was administered (22.5 mg/kg) to pregnant dams at embryonic day 9.5 (i.e., E9.5), which resulted in Arch-EGFP expression in Dbx1 neurons of their *Dbx1*^*CreERT2*^;Ai35D offspring.

### Respiratory active transverse slice preparations

Neonatal *Dbx1*^*CreERT2*^;Ai35D pups (postnatal days 0–4) were anesthetized by hypothermia and decerebrated. Mice were then dissected in 4°C artificial cerebrospinal fluid (aCSF) containing (in mM): 124 NaCl, 3 KCl, 1.5 CaCl_2_, 1 MgSO_4_, 25 NaHCO_3_, 0.5 NaH_2_PO_4_, and 30 dextrose. The aCSF was aerated continuously with carbogen (95% O_2_ and 5% CO_2_, pH 7.4). We removed the neuraxis, glued it to an agar block, and then cut 500-μm-thick transverse slices whose rostral surface exposed the border of the preBötC [22]. Slices were anchored in a recording chamber on a fixed-stage microscope and perfused with aCSF at 27°C at 2 ml·min^-1^. We recorded inspiratory-related motor output from hypoglossal (XII) nerve rootlets using a differential amplifier (gain 2000x) and a band-pass filter (300–1000 Hz). Nerve output was full-wave rectified and smoothed for display. Extracellular K^+^ in the aCSF was elevated to 9 mM to sustain robust rhythm and motor output [23,24].

We identified Dbx1 neurons by membrane-bound native EGFP expression (which does not fill the cytosol) and performed whole-cell patch-clamp recordings under visual control. Patch pipettes with tip resistance of 4–6 MΩ were fabricated from capillary glass (1.50 mm outer diameter, 0.86 mm inner diameter) and filled with solution containing (in mM): 140 potassium gluconate, 5 NaCl, 0.1 EGTA, 10 HEPES, 2 Mg-ATP, and 0.3 Na_3_-GTP. Alexa 568 hydrazide dye was added to the patch-pipette solution (50 μM, Invitrogen, Carlsbad, CA). Membrane potential was amplified (100x) and low-pass filtered (1 kHz) using a current-clamp amplifier (Dagan IX2-700, Minneapolis, MN) before being digitally acquired at 4 kHz (PowerLab 4/30, AD Instruments, Colorado Springs, CO).

### Surgery for optical fiber implantation

We anesthetized adult mice (aged 8–20 weeks) via intraperitoneal injection of ketamine (100 mg·kg^-1^) and xylazine (10 mg·kg^-1^) and performed aseptic surgeries in a stereotaxic frame. After exposing the skull, we performed two 0.5-mm-diameter bilateral craniotomies in the range 6.95 to 7.07 mm posterior to bregma and 1.15 to 1.5 mm lateral to the midline suture. In control animals, craniotomies were preformed 0.25 to 1.0 mm rostral to preBötC targeted locations. We joined 1.27-mm-diameter ceramic ferrules (Precision Fiber Products, Milptas, CA) with 105-μm-diameter 0.22 numerical aperture (NA) multimode fibers (Thorlabs, Newton, NJ) and implanted them 4.95 to 5.10 mm deep for preBötC experiments and 2.75 to 4.25 mm deep for control. Implants were secured using a cyanoacrylate adhesive (Loctite 3092, Westlake, OH) and anchored with a screw. Wounds were closed with a suture and tissue adhesive. The ferrule-fibers were connected to a 200-mW, 589-nm diode-pumped solid-state laser (Dragon Lasers, Chang Chun, China) using a line splitter and fiber coupler (OZ Optics, Ottawa, Canada). We recorded 2 min videos that incorporated 30-s bouts of light application to assess whether visible breathing movements would be affected by fibers implanted in preBötC or rostral to preBötC (e.g. S1 and S2 Videos). We injected ampicillin (4 mg·kg^-1^) and ketoprofen (125 mg·kg^-1^, s.c.) following surgery, and again 24 hr later, to manage pain and prevent infection. Mice recovered a minimum of ten days before further experimentation.

### Breathing measurements

After anesthetizing mice using 2% isoflurane we connected their ferrules to the 589-nm laser. Mice recovered from anesthesia for ~1 hr before we measured breathing behavior. Awake mice were placed unrestrained in a whole-body plethysmograph (Emka Technologies, Falls Church, VA). In a separate session, these same mice were lightly sedated via intraperitoneal ketamine injections (25 mg·kg^-1^ minimum dose) and titrated as needed to reduce limb movements but not abolish toe pinch and blink reflexes with a maximum aggregate dose of 50 mg·kg^-1^. Mice were then fitted with a nose cone (SOMNO-0801, Kent Scientific, Torrington, CT) for breathing measurements.

We applied a circuit of positive pressure, with balanced vacuum, to continuously flush the plethysmograph or nose cone with breathing air. A 1-liter respiratory flow head and differential pressure transducer (Spirometer, AD Instruments) measured airflow in all cases. Analog breathing signals were digitized at 1 kHz (PowerLab 4/30).

Bouts of illumination (2 s in duration) were applied during periods of restful wake over a 1-hr recording period. If a subject moved during a 5-s time window preceding, succeeding, or during the 2-s illumination phase, then these data were not analyzed to exclude movement-related artifacts superimposed in the breathing pattern. Light intensity measured 15 mW at the tissue contact point. Given the wavelength of light (589 nm), the diameter (105 μm) and NA (0.22) of the optical fiber, as well as an estimated distance of 0.75 mm from the fiber tip to distal edge of the preBötC (determined from histological sections), we used light dispersal and tissue scattering formulae [25,26] to calculate that preBötC neurons experienced an irradiance of not less than 12 mW·mm^-2^. Whole-cell recordings from hippocampal pyramidal neurons in mouse brain slices showed that 10 mW·mm^-2^ activates 60% of the available Arch-mediated outward current and evokes 83% of the maximum Arch-inducible hyperpolarization [21]. Those data, in combination with measurements and calculations above, suggest that our protocols deliver sufficient light to Dbx1 preBötC neurons to evoke close to saturating levels of Arch-mediated outward current.

### Histology

After the experiments we administered a lethal dose of pentobarbital (100 mg·kg^-1^, i.p.) to adult mice, which were then transcardially perfused with 0.1 M phospahte-buffered saline followed by 4% paraformaldehyde in 0.1 M phospahte-buffered saline. The neuraxes were removed and post-fixed overnight in 4% paraformaldehyde, and later sliced in 50-μm contiguous transverse sections. Free-floating sections were stained using 1% thionin acetate solution for 1 min, rinsed in distilled water, and finally washed in successive ethanol baths (2 min in 50% EtOH, 1 min in 75% EtOH, and then 2 min in 50% EtOH twice). Slices were mounted on gelatin coated slides and dehydrated using four graded ethanol baths (3 min in 70% EtOH, 5 min in 90% EtOH, and 5 min in 100% EtOH twice), cleared using four xylenes immersions (one for 30 s followed by three for 5 min), and then cover-slipped using DPX (Sigma-Aldrich, St. Louis, MO). Tissue sections were visualized using bright-field microscopy. Images were arranged as mosaics and brightness and contrast were adjusted uniformly across the entire ensemble image using the public domain software package ImageJ [27].

### Data analysis

The airflow signal was band-pass filtered (0.1 to 20 Hz) and analyzed using LabChart 7 software (AD Instruments). From filtered airflow traces using the spirometry module in LabChart 7 we calculated tidal volume (V_T_), inspiratory duration (T_i_), and respiratory rate (*f*_R_). Minute Ventilation (MV) was calculated automatically by LabChart 7 by multiplying the average V_T_ and *f*_R_ within a 0.5-s rolling time window. We employed a 0.5-s time window centered on peak inspiratory airflow to compute cycle-triggered averages (CTA) prior to and during bouts of light presentation. Peak airflow was detected via a local maxima-detecting algorithm and CTAs were calculated using algorithms built into LabChart 7. We computed statistics using Graphpad Prism 6 (La Jolla, CA) and prepared figures using Adobe Illustrator (Adobe Systems Inc., San Jose, CA) as well as IGOR Pro 6 (Wavemetrics, Lake Oswego, OR). Group data are reported as mean ± standard deviation (SD). We employed the Friedman Test, which is a non-parametric statistical test comparable to a one-way ANOVA, for statistical hypothesis testing.

## Results

### Light-evoked hyperpolarization of Dbx1 preBötC neurons interrupts inspiratory rhythm and motor output in vitro

**Fig 1:**
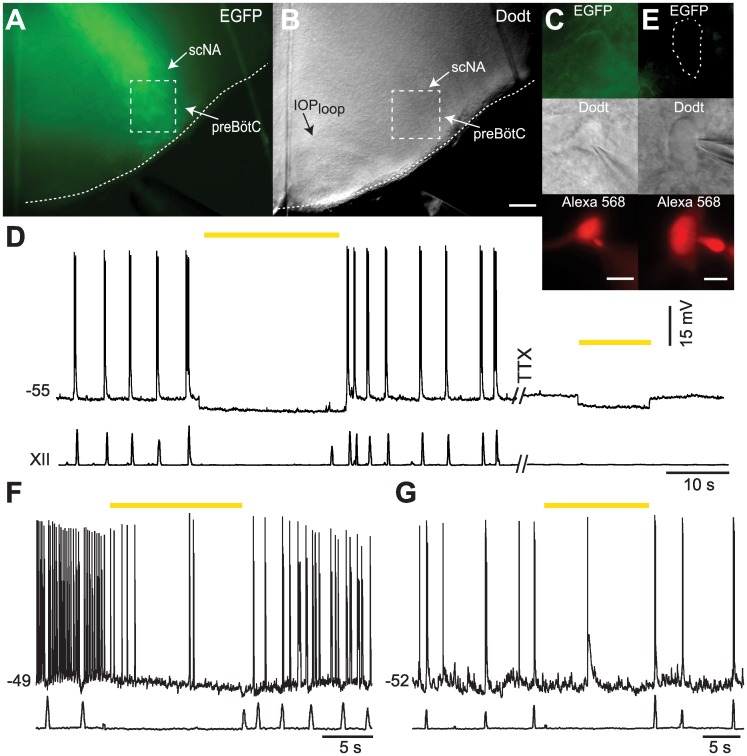
Light activation of Arch-expressing Dbx1 preBötC neurons hyperpolarizes Dbx1 neurons and precludes respiratory rhythm. **A**, Rostral slice surface of a P2 *Dbx1*^*CreERT2*^;Ai35D mouse showing Arch-GFP expression. Dotted box marks the preBötC. **B**, Dodt image of the slice in A showing location of the preBötC relative to known anatomical markers, the principal loop of the inferior olive (IOP_loop_) and semicompact division of the nucleus ambiguus (scNA). The scale bar represents 150 μm and applies to both A and B. **C**, Inspiratory Dbx1 neuron visually identified by membrane-delimited EGFP expression (top), Dodt contrast microscopy (middle), and by dialysis of Alexa Fluor 568 introduced via the patch pipette solution after the onset of whole-cell recording (bottom). Scale bar represents 10 μm. **D**, Membrane potential trajectory of the neuron in C with synchronous XII output. TTX was applied at 1 μM. Voltage and time calibrations are shown **E**, A non-Dbx1 neuron lacking EGFP expression (top), identified in Dodt contrast microscopy and via Alexa Fluor 568 dialysis (bottom). Scale bar represents 10 μm. **F**, Membrane potential trajectory of the non-Dbx1 preBötC neuron in E with synchronous XII output. **G**, Membrane potential trajectory of a non-Dbx1 neuron with inspiratory modulation. Voltage calibration in D applies to F and G; separate time calibrations are shown. 589-nm light application is indicated by yellow bars in D, F, and G.

Transverse medullary slices from neonatal mice that retain the preBötC spontaneously generate inspiratory rhythm and XII motor output. *Dbx1*^*CreERT2*^;Ai35D mouse slices show native EGFP expression continuously from the XII nucleus dorsomedially, through the intermediate reticular formation and preBötC, to the ventrolateral border (Fig 1A). We recorded Dbx1 neurons ventral to the semi-compact division of the nucleus ambiguus and orthogonal to the dorsal boundary of the principal loop of the inferior olive (Fig 1B), which coincides with the rostral face of the preBötC [22]. Exposure to 589-nm light hyperpolarized the baseline membrane potential of five Dbx1 preBötC neurons by 5.6 ± 1.8 mV, which persisted in the presence of 1 μM tetrodotoxin (TTX) (Fig 1C and 1D).

In contrast, light exposure had a negligible impact on the baseline membrane potential of six non-Dbx1 preBötC neurons that showed either expiratory (Fig 1E and 1F) or non-respiratory firing patterns, as well as non-Dbx1 preBötC neurons with evidence of inspiratory modulation (Fig 1G). The average change in baseline membrane potential for all non-Dbx1 neurons measured –0.68 ± 0.3 mV.

Illumination of the preBötC in 30-s bouts generally stopped XII output (26 total bouts in four slices). However, a single attenuated XII burst occurred in the last 10 s of the 30-s bout in four instances across three slices (e.g., Fig 1D).

These results indicate that activation of Arch hyperpolarizes Dbx1 preBötC neurons not via network disfacilitation but rather direct postsynaptic effects. In neonatal mouse slices, the majority of Dbx1 preBötC neurons are rhythmogenic, expect perhaps those that express the peptide transmitter somatostatin [19]. A subset of Dbx1 neurons at the dorsal edge of the preBötC have premotor functionality [28,29], but far more XII premotor neurons are located in the intermediate reticular formation, adjacent dorsally to the preBötC [28,30]. Therefore, in the present context, bilateral illumination of the preBötC most likely stops rhythmic XII output via suppression of rhythmogenesis rather than premotor blockade.

### Arch-mediated inhibition in Dbx1 preBötC neurons suppresses breathing in anesthetized and sedated mice

**Fig 2:**
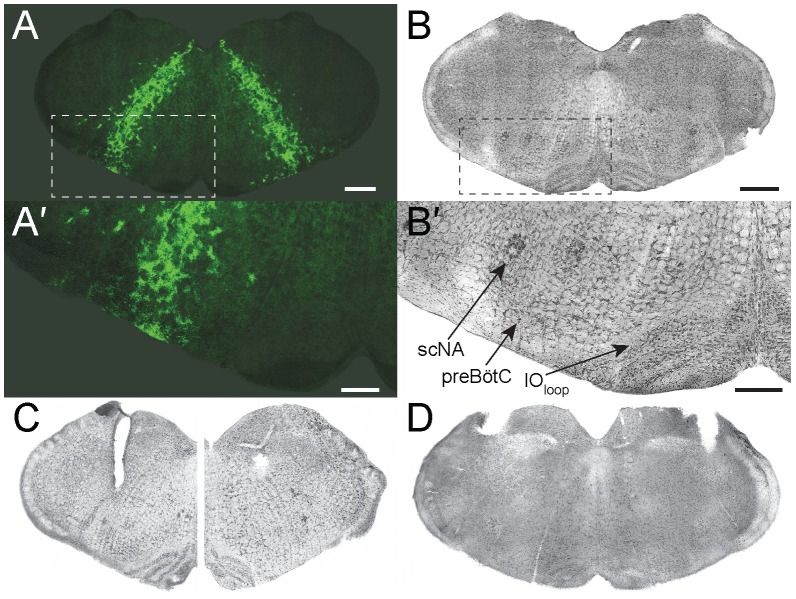
Arch-EGFP expresion and histology of fiber-optic implants in adult *Dbx1*^*CreERT2*^;Ai35D mice. **A**, EGFP expression in 35 week-old *Dbx1*^*CreERT2*^;Ai35D mouse. Scale bar represents 500 μM. **A´**, Inset of boxed region in A showing an expanded view of the ventral region of the slice, which includes the preBötC. Scale bar represents 250 μM. **B**, Bright field image of a thionin-stained section adjacent to A. Scale bar represents 500 μM and applies to B, C, and D. **B´**, Inset of boxed region in B showing an expanded view of the ventral region of the slice, which shows visible markers that co-locate with the preBötC including the semicompact division of the nucleus ambiguus (scNA) and the principal loop of the inferior olive (IO_loop_). Scale bar represents 250 μM. **C**, Bright field images of adjacent thionin-stained sections from an experimental mouse whose fiber-optics and ferrules targeted the preBötC. **D**, Bright field image of thionin-stained section from an experimental mouse whose fiber-optics and ferrules targeted medullary circuitry dorsal and rostral to the preBötC.

We examined Arch-EGFP expression in adult *Dbx1*^*CreERT2*^;Ai35D mice, which recapitulated the pattern characterized by Dbx1-reporter expression in developing embryos and neonates [14,15,22]. Viewed in transverse sections, Dbx1-derived cells form an inverted V-shaped pattern extending from the XII nucleus dorsomedially to the ventrolateral border of the section, which incorporates the intermediate reticular formation and the ventral respiratory column (Fig 2A). The position of the preBötC can be determined from anatomical markers including the principal loop of the inferior olive (IO_loop_), the semi-compact division of the nucleus ambiguus (scNA), and the shallow U-shape of the fourth ventricle, which indicates proximity to the obex (Fig 2B and 2B’) [31].

We implanted fiber optics bilaterally to activate Arch in Dbx1 preBötC neurons in vagus-intact adult *Dbx1*^*CreERT2*^;Ai35D mice (positions confirmed post-hoc, e.g., Fig 2C). Control littermates had fiber optics implanted dorsally in the medulla at a position rostral to the preBötC (e.g., Fig 2D). Immediately after implantation we delivered 589-nm laser pulses (30 s) while visually monitoring breathing. Anesthetized mice whose ferrules were implanted in the preBötC transiently stopped ventilation for intervals of approximately 18 s (n = 6, S1 Video), whereas breathing remained unperturbed in control mice with ferrules in the dorsal medulla (n = 7, S2 Video).

**Fig 3:**
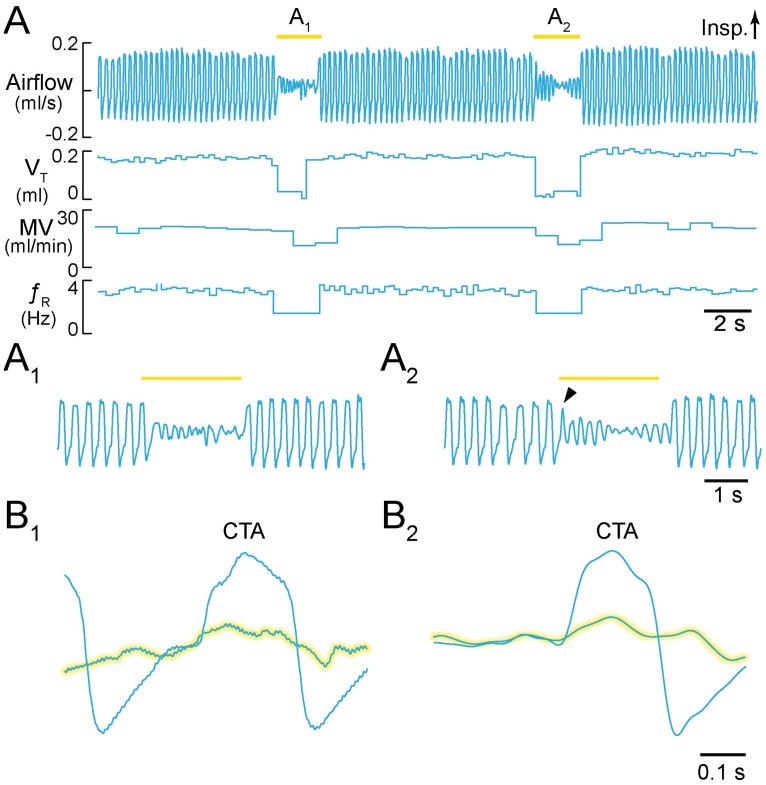
Activation of Arch in Dbx1 preBötC neurons in sedated mice. **A**, Airflow, V_T_, MV, and *f*_R_ plotted continuously during two consecutive 2-s light pulses. Inspiratory airflow is plotted upward, which reflects nose-cone measurements. **A**_**1**_, **A**_**2**_, Expanded airflow traces from A. Arrowhead in A_2_ indicates light-evoked interruption of the inspiratory phase. Yellow bars indicate 589-nm light application. **B**_**1**_, **B**_**2**_, Cycle-triggered averages of airflow from each bout prior to (not highlighted) and during illumination (highlighted). Time calibrations are shown for each panel.

After ten days of recovery, we measured breathing via a nose cone in six lightly ketamine-sedated *Dbx1*^*CreERT2*^;Ai35D mice. Prior to preBötC illumination, these mice breathed at a *f*_R_ of 2.9 ± 0.5 Hz, with V_T_ of 0.12 ± 0.08 ml, MV of 21.8 ± 14.9 ml·min^-1^, and T_i_ of 130 ± 30 ms. During 2 s of preBötC illumination, *f*_R_ decreased to 1.5 ± 0.9 Hz (p = 0.0001), V_T_ decreased to 0.07 ± 0.04 ml (p = 0.0001), and MV decreased to 9.9 ± 5.6 ml·min^-1^ (p = 0.0001). In contrast, T_i_ increased to 280 ± 90 ms (p = 0.008) during preBötC illumination (Fig 3A).

We examined the airflow fluctuations during bouts of illumination (Fig 3A_1_ and 3A_2_). Inspiratory movements discontinued at the onset of light application (e.g., Fig 3A_2_ arrowhead) followed by damped airflow fluctuations. Cycle-triggered averaging showed these airflow fluctuations to be aperiodic and attenuated in amplitude, which indicates respiratory ataxia (Fig 3B_1_ and 3B_2_).

**Fig 4:**
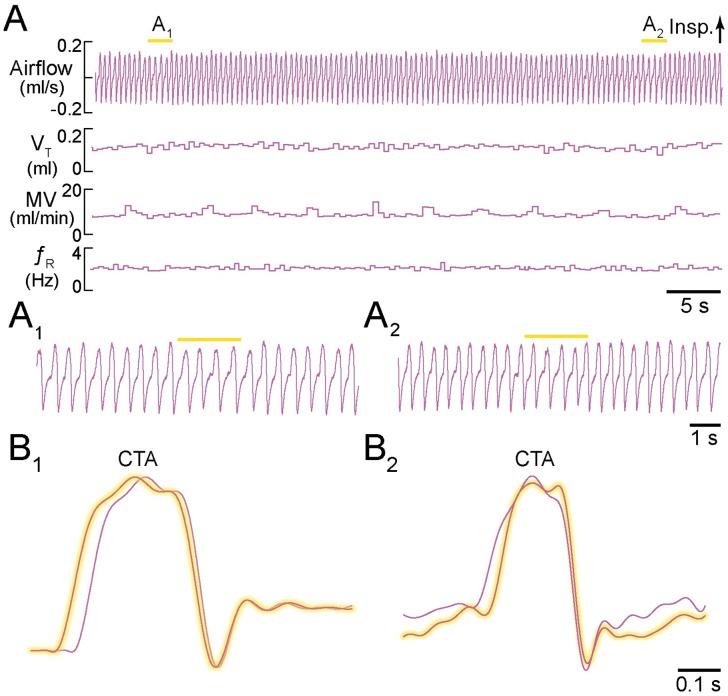
Light application to the dorsal medulla rostral to preBötC in sedated mice. **A**, Airflow, V_T_, MV, and *f*_R_ plotted continuously during two consecutive applications of 2-s light pulses. Inspiratory airflow is plotted upward, which reflects nose-cone measurements. **A**_**1**_, **A**_2_, Expanded airflow traces from A. Yellow bars indicate 589-nm light application. **B**_**1**_, **B**_**2**_, Cycle-triggered averages of airflow from each bout prior to (not highlighted) and during illumination (highlighted). Time calibrations are shown for each panel.

Illumination of the dorsal medulla in seven lightly ketamine-sedated *Dbx1*^*CreERT2*^;Ai35D controls (positions confirmed post-hoc, e.g., Fig 2D) did not modify breathing. Prior to illumination of the dorsal medulla, *f*_R_ measured 3.3 ± 0.7 Hz, with V_T_ of 0.12 ± 0.12 ml, MV of 14.4 ± 14.1 ml·min^-1^, and T_i_ of 140 ± 20 ms. During bouts of illumination, *f*_R_ measured 3.3 ± 0.7 Hz (p = 0.30), with V_T_ of 0.11 ± 0.12 ml (p = 0.62), MV of 13.9 ± 13.2 ml·min^-1^ (p = 0.2), and T_i_ of 140 ± 20 ms (p = 0.97) (Fig 4A). Cycle-triggered averages of respiratory airflow prior to and during illumination were virtually superimposable (Fig 4B).

### Arch-mediated inhibition in Dbx1 preBötC neurons perturbs breathing in awake mice

**Fig 5:**
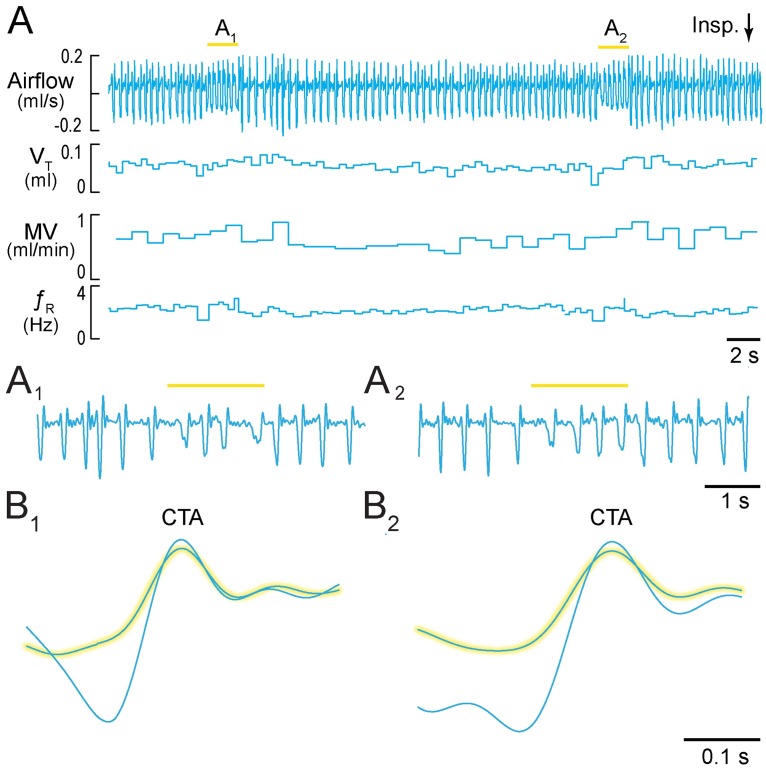
Activation of Arch in Dbx1 preBötC neurons in freely behaving awake mice. **A**, Airflow, V_T_, MV, and *f*_R_ plotted continuously during two consecutive applications of 2-s light pulses. Inspiratory airflow is plotted downward, which reflects whole-body plethysmography. **A**_**1**_, **A**_**2**_, Expanded airflow traces from A. Yellow bars indicate 589-nm light application. **B**_**1**_, **B**_**2**_, Cycle-triggered averages of airflow from each bout prior to (not highlighted) and during illumination (highlighted). Note that inspiratory airflow is attenuated, whereas expiratory airflow is not. Time calibrations are shown for each panel.

In the same cohort of awake and unrestrained adult *Dbx1*^*CreERT2*^;Ai35D mice, we illuminated the preBötC and monitored breathing via whole-body plethysmography. Light delivery to the preBötC decreased *f*_R_ (from 2.3 ± 0.6 in control to 2.1 ± 0.5 Hz during preBötC illumination, p = 0.0012) without modifying V_T_ (0.05 ± 0.008 vs. 0.05 ± 0.001 ml, p = 0.24) or MV (0.87 ± 0.1 vs. 1.0 ± 4.7 ml·min^-1^, p = 0.49; because of two outliers in the ‘Light’ condition we report MV in the text using the median ± SD (rather than the mean ± SD). Illumination of the preBötC also increased T_i_ from 100 ± 20 ms to 120 ± 11 ms (p = 0.0012) and decreased inspiratory airflow with no concommitant effect on expiration (Fig 5A). The decrease in *f*_R_, reduced inspiratory amplitude, and longer T_i_ during illuminated cycles are illustrated more clearly at faster sweep speed (Fig 5A_1_ and 5A_2_) and in cycle-triggered averages (Fig 5B_1_ and 5B_2_).

**Fig 6:**
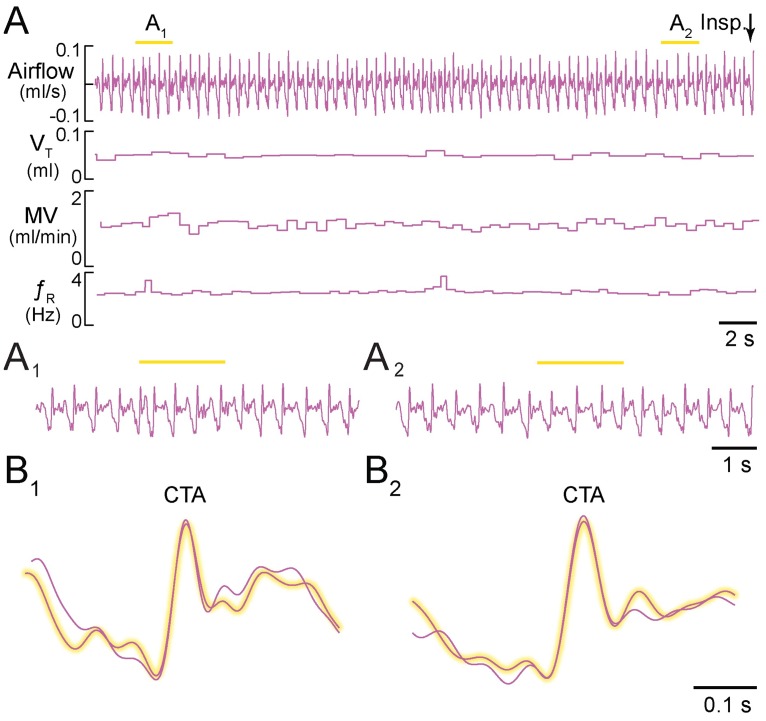
Light application to the dorsal medulla rostral to preBötC in freely behaving awake mice. **A**, Airflow, V_T_, MV, and *f*_R_ plotted continuously during two consecutive applications of 2-s light pulses. Inspiratory airflow is plotted downward, which reflects whole-body plethysmography. **A**_**1**_, **A**_**2**_, Expanded airflow traces from A. Yellow bars indicate 589-nm light application. **B**_**1**_, **B**_**2**_, Cycle-triggered averages of airflow from each bout prior to (not highlighted) and during illumination (highlighted). Time calibrations are shown for each panel.

**Fig 7:**
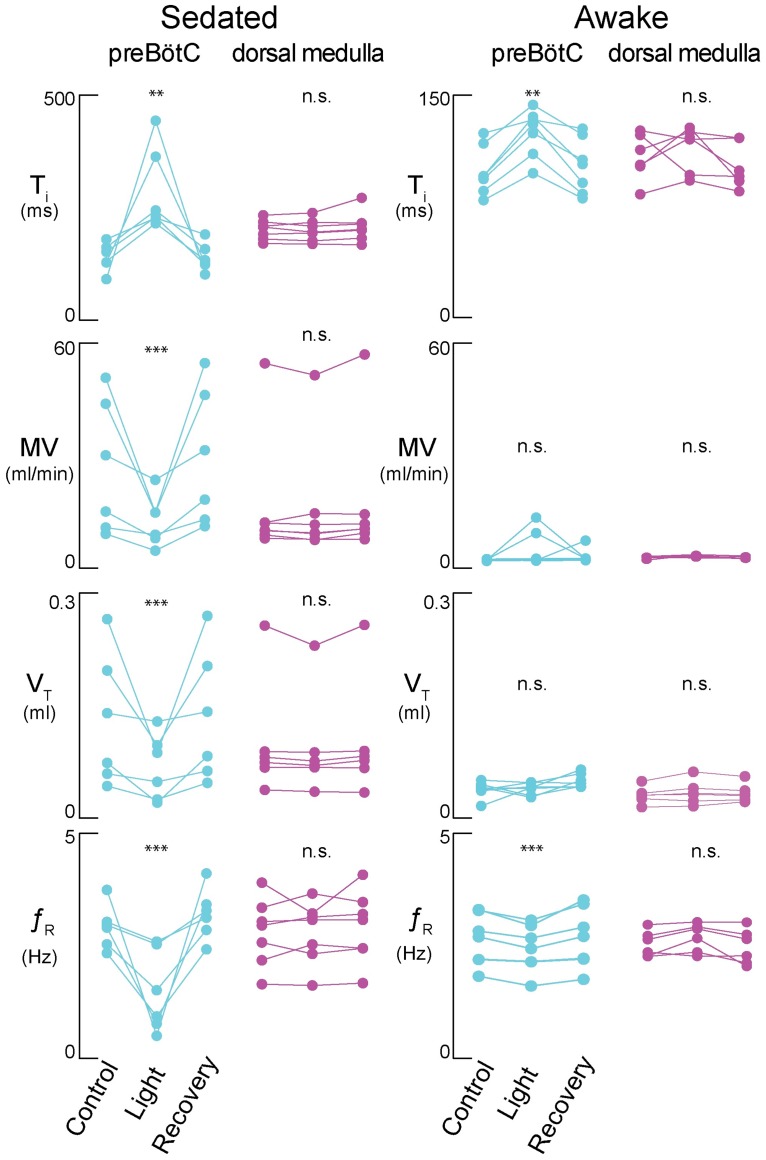
The respiratory effects of Arch-mediated photoinhibition. The left column shows effects in sedated animals. The right column shows effects in freely-behaving awake animals. Cyan symbols pertain to illumination of the preBötC whereas magenta symbols pertain to illumination of the dorsal medulla rostral to preBötC. Respiratory measurements include T_i_ (first row), MV (second row), V_T_ (third row), and *f*_R_ (fourth row). Control, light application, and recovery data are shown for all experimental subjects. Double asterisks refer to the probability of a type I statistical error with alpha < 0.01. Triple asterisks refer to the probability of a type I statistical error with alpha < 0.001. “n.s.” (i.e., not significant) refers to the probability of a type I statistical error with alpha > 0.05.

Illumination of the dorsal medulla in seven *Dbx1*^*CreERT2*^;Ai35D mice had no notable effect on respiration. Prior to illumination of the dorsal medulla, *f*_R_ measured 2.6 ± 0.3 Hz, with V_T_ of 0.06 ± 0.01 ml, MV of 1.00 ± 0.12 ml·min^-1^ and T_i_ of 120 ± 150 ms. During bouts of illumination, *f*_R_ measured 2.8 ± 0.3 Hz (p = 0.18, n = 7), with V_T_ of 0.06 ± 0.15 ml (p = 0.25), MV of 1.10 ± 0.11 ml·min^-1^ (p = 0.429), and T_i_ of 120 ± 15 ms (p = 0.25) (Fig 6A). The breathing pattern remained unchanged during cycles of illumination (Fig 6A), which was clear at faster sweep speed (Fig 6A_1_ and 6A_2_) and in cycle-triggered averages (Fig 6B_1_ and 6B_2_). All of the group data are shown in Fig 7.

## Discussion

According to the Dbx1 core hypothesis, interneurons derived from Dbx1-expressing precursors comprise the CPG for inspiratory breathing movements. We investigated this hypothesis in vagus-intact adult mice using intersectional mouse genetics to express Arch in Dbx1 neurons, and then perform acute optogenetic silencing while monitoring its respiratory effects.

Evidence from studies in perinatal mice favored the Dbx1 core hypothesis at the outset of this investigation. Dbx1 neurons in the ventral medulla express glutamate and peptide neuromessengers, as well as peptide receptors, which are characteristics closely aligned with respiratory rhythmogenic function [3–7,9,10,32,19]. The commissural axons of Dbx1 neurons synchronize preBötC rhythms bilaterally and no recognizable preBötC forms in *Dbx1* knock-out mice, which die at birth of asphyxia [14,15]. Furthermore, laser ablation of Dbx1 preBötC interneurons in neonatal slices ultimately precludes respiratory rhythm and motor output [16]. Therefore, it was not surprising that bilateral illumination of the preBötC in *Dbx1*^*CreERT2*^;Ai35D mouse slice preparations hyperpolarized Dbx1 neurons and arrested rhythmic XII output. Koizumi *et al*. reported similar data in reduced in situ preparations as well as mouse slices using a different Dbx1 Cre-driver strain but the same floxed-Arch reporter [18].

We interpret these results to mean that hyperpolarizing Dbx1 preBötC neurons directly impedes core rhythmogenic function, an interpretation equally advocated by Koizumi *et al*. [18]. One potential caveat is that Arch-EGFP expression is not constrained to the cell bodies of Dbx1 neurons, so one must consider photoinhibition of axons and axon terminals. Dbx1 neurons are found throughout the respiratory medulla [22] and some likely project to the preBötC. Axon terminals with remote origin that express optogenetic fusion proteins remain viable in transverse slices from the respiratory medulla [33]. Therefore, illumination of Arch-EGFP-expressing axon terminals could conceivably disfacilitate the preBötC to impede rhythmogenesis.

While we cannot rule out this interpretation, slice experiments provide negligible evidence of disfacilitation. Light application hyperpolarized Dbx1 neurons by ~6 mV in the context of network activity and in TTX, which is consistent with direct postsynaptic photoinhibition (Fig 1). We presume that Dbx1 and non-Dbx1 neurons in the preBötC, both of which exhibit inspiratory rhythmic behavior and have similar membrane properties [17], receive commensurate sources of tonic drive. That drive could originate (at least in part) from Dbx1 neurons with distant somata whose Arch-EGFP-expressing axon terminals synapse in the preBötC. However, illumination resulted in less than 1 mV of hyperpolarization in non-Dbx1 neurons (that do not express Arch-EGFP and thus cannot experience postsynaptic photoinhibition), which suggests that light-evoked disfacilitation inappreciably influences baseline membrane potential and excitability in Dbx1 as well as non-Dbx1 preBötC neurons.

We cannot yet noninvasively monitor neural activity in the preBötC of awake intact adult mice to ascertain whether photoinhibition silences Dbx1 neurons or simply diminishes their activity. Nevertheless, if Dbx1 preBötC neurons are rhythmogenic in intact adult mice then straightforward predictions are that photoinhibition should either: i) cause apnea or ii) retard the progress of the inspiratory phase and thus prolong inspiratory duration (if breathing persists during bouts of preBötC illumination), as well as lengthen the interval between inspiratory phases (i.e., decrease *f*_R_). Continuous laser pulses that affect the respiratory cycle in its entirety caused all of the predicted effects: apnea, prolonged T_i_, and decreased *f*_R_.

Anesthetized mice experienced 18-s apneas during 30-s bouts of preBötC illumination (S1 Video) in accord with the first prediction. Sedated mice transiently increased T_i_ and decreased *f*_R_, V_T_, and MV during 2-s bouts of preBötC illumination (Figs 3 and 7). The effects on T_i_ and *f*_R_ match the second prediction (Fig 7). The decrease in V_T_ and MV reflect the reduced *f*_R_ as well as smaller amplitude inspiratory breaths, and remain consistent with the second prediction.

We analyzed the airflow signal during preBötC illumination in sedated mice as if it represented an attenuated preBötC respiratory rhythm; diminished *f*_R_, V_T_, and MV, combined with prolonged T_i_ are consistent with this explanation. However, the reduced-amplitude airflow fluctuations during light application were aperiodic according to the CTAs. Therefore, it remains possible that illumination precludes a preBötC-driven respiratory rhythm and that the attendant airflow fluctuations reflect a non-preBötC behavior that nonetheless affects airflow. In support of this idea, the onset of the laser pulse halted inspiratory efforts mid-cycle (e.g., Fig 1A_2_). Whisking or other orofacial behaviors could register airflow if the preBötC were offline. The whisking CPG is adjacent to the preBötC but employs disparate cellular and synaptic mechanisms [34,35] that unlikely to be affected by photoinhibition of Dbx1 neurons.

Whether or not preBötC-generated, airflow fluctuations during bouts of preBötC illumination would not ventilate the mouse. V_T_ was attenuated by ~50% (Fig 3A_1_ and 3A_2_). This volume is insufficient to clear the dead space associated with airways and trachea, which constitutes 30–45% of V_T_ in mammals ranging from rodents to horses [36,37] and 19–30% of vital capacity in mice [38].

Illuminating the preBötC in awake intact adult mice transiently decreased *f*_R_ and increased T_i_ combined with lowered ampitude inspiratory breaths. However, there was no change in V_T_ or MV. The effects on *f*_R_, T_i_, and inspiratory breath amplitude are consistent with suppressing the preBötC core oscillator, but the lack of effect on V_T_ and MV is at odds with that interpretation. If the Dbx1 core hypothesis is true, then why does ventilation persist in intact adult mice?

Perhaps the light-evoked outward current in Dbx1 preBötC neurons is insufficient to suspend rhythmogenesis. According to our measurements and calculations, the light intensity at the preBötC was adequate to evoke near maximum Arch-mediated current (see Materials and Methods). However, if Arch-EGFP expression were limited within the Dbx1 preBötC neuron population, then (regardless of light delivery), it could diminish the potential for optogenetic suppression of respiratory rhythmogenesis.

Dbx1 is expressed between E8.5 and E12.5 [13–15]. We activated CreER^T2^ at E9.5 when we presume *Dbx1* expression peaks. Thus, Cre-Lox recombination will not occur in the fraction of Dbx1-expressing precursors that enter mitosis prior to E9.5. Furthermore, CreER^T2^ recombination is inherently fragmentary, so one expects Arch-EGFP underexpression in the target population.

Even if we stipulate ideal Arch-EGFP expression and light delivery, optogenetic suppression of respiration in awake intact mice may not be feasible because of excitatory drive and sensory feedback. Chemosensitive neurons in the retrotrapezoid nucleus [39] as well as excitatory inputs from the pons and raphé [40–42] tonically excitate the preBötC. Furthermore, with the vagus nerve intact, lung inflation and deflation reflexes maintain high *f*_R_ and limit T_i_ (generally 2–4 Hz and ~100 ms, respectively, in mice). Vagotomy reduces respiratory frequency by 50–65% and extends inspiratory duration two-fold in rodents [43,44]. Therefore, sources of tonic excitation and sensory feedback may override the ~6 mV of light-evoked hyperpolarization in some fraction of the Dbx1 preBötC neuron population such that photoinhibition impedes but does not stop rhythmogenesis nor inspiratory breathing movements. In support of this idea that tonic sources ofdrive can override Arch effects, optogenetic inhibition of Dbx1 preBötC neurons was unable to stop fictive respiratory rhythms in a completely deafferented adult in situ preparation, except when the medulla was transversely transected at the medullary junction rostral to the preBötC, which would abolish all sources of tonic drive [18].

Arch-mediated photoinhibition probably provides a stronger impediment to breathing in anesthetized and sedated mice because drugs, notably ketamine and ketamine-xylazine, generally suppress respiration [45–47], which would act in concert with Arch.

Alternatively, it is conceivable that the respiratory core oscillator in adults incorporates non-Dbx1-derived interneurons, which are not active perinatally and in adults would remain unperturbed by 589-nm light. One candidate population in the ventral medulla would be catecholamanergic C1 neurons, which also utilize glutamate as a fast transmitter [48,49]. However, these neurons are associated with autonomic regulation, particularly circulation at the level of the rostral ventrolateral medulla. Although optogenetic excitation of C1 neurons modulates respiratory rhythm in conscious mice, it does so in a cardiorespiratory integrative context [50]. Furthermore, C1 neurons do not express neurokinin-1 receptors [5] nor do they form commissural projections onto preBötC neurons, which are hallmark features of respiratory rhythmogenic preBötC neurons. Therefore, we think it unlikely that C1 neurons contribute a heretofore unidentified respiratory rhythmogenic circuit.

Although we cannot rule out the existence of non-Dbx1 interneurons that sustain rhythmogenesis during photoinhibition of Dbx1 preBötC neurons, the most parsimonious explanation for persistent ventilation in intact adult *Dbx1*^*CreERT2*^;Ai35D mice during preBötC illumination is that excitatory drive from modulatory and chemosensitive inputs, as well as vagal sensory feedback, provide sufficient excitation to Arch-EGFP-expressing Dbx1 preBötC neurons to overcome light-mediated inhibition and sustain ventilation. Nevertheless, photoinhibition impedes core rhythmogenic function, resulting in inspiratory breaths at lower amplitude and frequency, with prolonged inspiratory duration.

We can attribute rhythmogenic function to Dbx1-derived interneurons in the preBötC [18,19], but neurons from the same genetic class serve in other respiratory and non-respiratory functions as well. For example, Dbx1 neurons at the dorsal edge of the preBötC, and in the intermediate reticular formation have premotor function related to inspiratory movements of the tongue muscle genioglossus [16,28]. Therefore, it may be possible to further subdivide Dbx1 preBötC neurons on the basis of genetic variation [51,52] to discern a specifically rhythmogenic subset.

## Supporting Information

S1 Video Activation of Arch using 589-nm light in Dbx1 preBötC neurons transiently arrests breathing in an anesthetized vagus-intact adult mouse. Video playback speed is 2x real time.
(MP4)

S2 Video Application of 589-nm light to the dorsal medulla rostral to the preBötC does not visibly affect breathing. Video playback speed is 2x real time.
(MP4)
